# A case series of Sneddon syndrome: clinical features, diagnostic workup, and literature review

**DOI:** 10.1186/s12883-025-04335-w

**Published:** 2025-11-28

**Authors:** Bruno Henrique Carneiro Costa Filho, Victor Ting Po Chy, João Augusto de Macedo Cavalcanti de Albuquerque, Pedro Jatobá Arteiro, Davi Alexandre Soares Neves, Mário Luciano de Mélo Silva Júnior, Eduardo Sousa de Melo

**Affiliations:** 1 Hospital das Clínicas da Universidade Federal de Pernambuco, ML Mélo Silva Jr, 1235 Moraes Rego Av, Cidade Universitária, Recife, 50670-901, Brazil; 2 Medical Science Center, Universidade Federal de Pernambuco, Recife, Brazil; 3 Medical Science Center, Area of Neuropsychiatry, Universidade Federal de Pernambuco, Recife, Brazil; 4 Neurology Unit, Hospital da Restauração, Recife, Brazil

**Keywords:** Sneddon syndrome, Antiphospholipid syndrome, Livedo racemosa, Stroke in young adults

## Abstract

**Background:**

Sneddon syndrome, a rare, non-inflammatory thrombotic vasculopathy characterized by livedo racemosa and cerebrovascular disease.

**Case presentation:**

We present a case series of six women diagnosed with Sneddon syndrome. We conducted a thorough analysis of clinical, radiological, and laboratory data, including results of prothrombotic and autoimmune screening. Our findings emphasize the importance of considering Sneddon syndrome as a potential cause of stroke, particularly in young women, and underscore the necessity of a comprehensive dermatological examination when evaluating stroke etiology. Additionally, we provide a comprehensive literature review of the clinical manifestations, radiological and histopathological findings, as well as treatment options.

**Conclusion:**

A thorough dermatological examination can aid in early detection of Sneddon syndrome and may change the course of treatment of stroke in young adults.

## Introduction

Sneddon syndrome (SS) is a non-inflammatory thrombotic vasculopathy, clinically characterized by the combination of livedo *racemosa* (LR), an erythematous-violaceous skin lesion with an irregular net-like pattern, and cerebrovascular disease [1]. In 1965, Ian Bruce Sneddon described six patients who had a history of multiple ischemic cerebral events and the presence of extensive skin lesions compatible with LR, but which were not explained by rheumatologic, hematologic, or infectious etiologies [2]. It remains a rare condition, with an estimated incidence in the general population of 4 cases per million persons per year, and occurs more frequently in women in the third to fifth decades of life [1, 3].

This study aims to present a case series of six women with SS under the care of our outpatient Neurology Unit specialized in cerebrovascular disorders and review the literature on this rare disease. We reviewed relevant clinical (age at stroke onset, vascular territory affected, neurologic deficits at presentation, obstetric history and other manifestations such as headache and epilepsy), radiologic and laboratory (prothrombotic and autoimmune screening results, as antiphospholipid syndrome [APS] and systemic lupus erythematosus [SLE] antibodies) data.

## Case reports

We diagnosed primary SS when a patient had at least one ischemic cerebral event and skin lesions compatible with LR, with no other identifiable etiological factors after thorough evaluation. In patients with coexisting systemic autoimmune diseases or a thrombophilic condition, we classified the case as secondary SS.

We present the case reports of 6 women with SS. Median age at presentation was 28.5 years old (IQR 22–39). Migraine with aura occurred in 50% of cases (3 out of 6). Furthermore, 3/6 (50%) were positive for at least one APS antibody. All patients were treated with warfarin, and did not present recurrence of stroke or complications in a median follow-up period of 5.5 years. Secondary SS was found in 5/6 (83.3%) of our cases.

Table 1 summarizes the data from all patients.

**Table 1:** Summary of neurological features, comorbidities and obstetric history of our reported patients

Case	Age at stroke event/s	Neurological features			Comorbidities				Obstetric history
		Headache, type	Epilepsy	Cognitive dysfunction	Positive APSa	SLE	SAH	Other findings	Gravida/para/abortus
#1	55	No	Yes, focal	Yes	ACL, LA	No	Yes	Overweight	G2P2A0
#2	19 and 20	Yes, tension type	No	No	No	No	No	Raynaud’s	G1P1A0
#3	39	Yes, MA	No	No	No	Yes	Yes	Mitral valve thickening	G4P2A2
#4	31	Yes, MA	No	No	Aβ2GP1	No	No	High titer ANA	G1P1A0
#5	26	Yes, MA	No	No	Aβ2GP1, ACL, LA	No	No	Raynaud’s, mitral valve insufficiency	G0A0P0
#6	22	No	Yes, focal	No	No	Yes	No	Overweight, antithrombin III deficiency	G0P0A0

*ACL* Anticardiolipin antibodies, *ANA* Antinuclear antibody, *APSa* Antiphospholipid syndrome antibodies, *Aβ2GP1* Anti-beta2-glycoprotein 1 antibodies, *LA* Lupus anticoagulant, *MA* Migraine with aura, *SAH* Systemic arterial hypertension, *SLE* Systemic lupus erythematosus

## Case 1

**Fig. 1:**
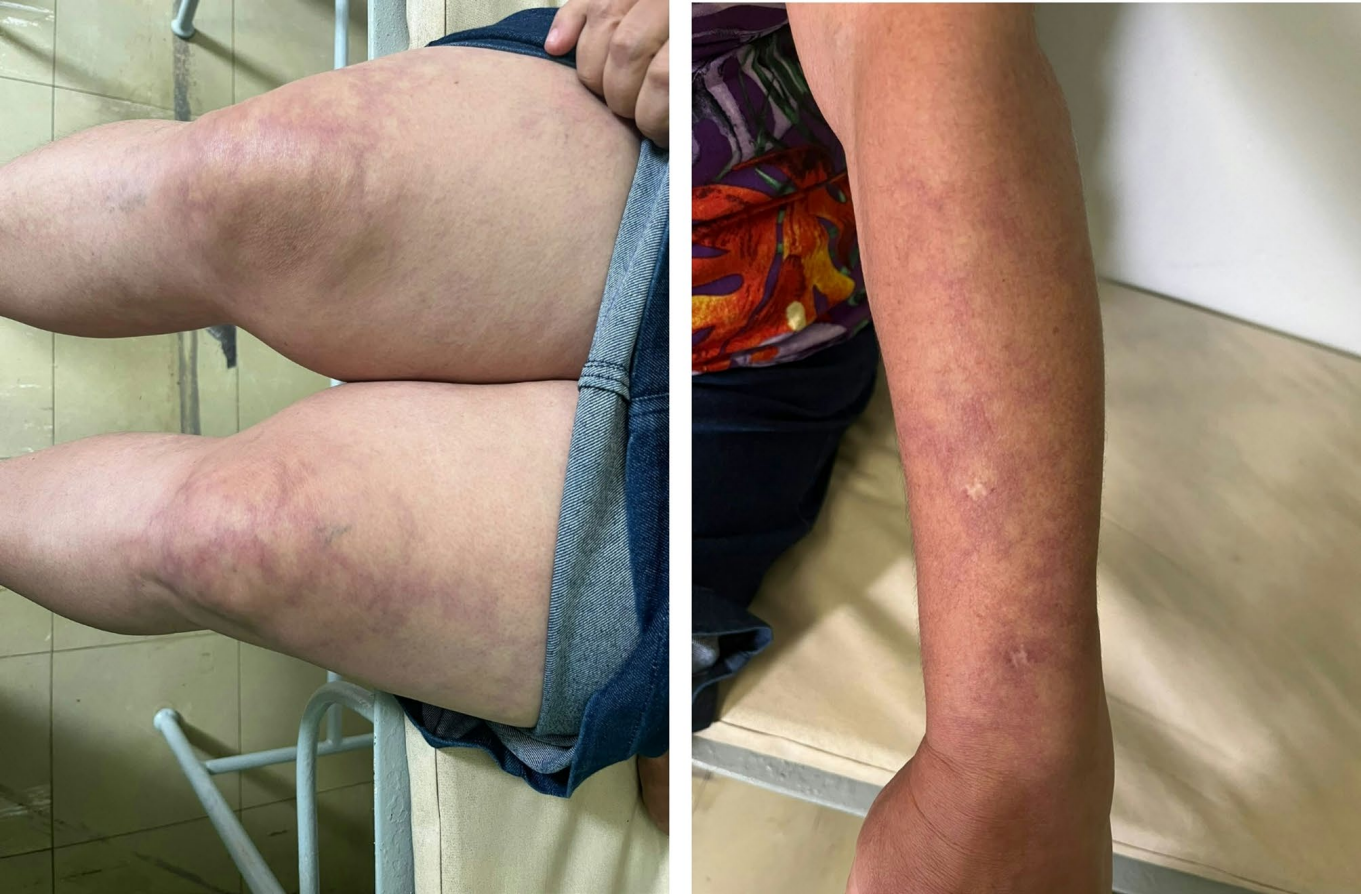
From Patient #1. Right: Purple and erythematous skin lesions, consistent with LR, are distributed on both thighs and knees, with irregular shapes and variable sizes. Larger confluent areas are present alongside smaller, more discrete patches. The increased concentration on the knees reflects the tendency for these lesions to develop in response to colder temperatures. Left: The same pattern of lesions can be seen on the dorsal aspect of the patient’s wrist extending to the mid-forearm

A woman developed epilepsy at 41 years-old, presenting with focal motor seizures. These events were well controlled with the use of carbamazepine. During that period, she already had LR (Fig. 1). A biopsy of the skin lesion was performed, yielding inconclusive results. Twelve years later, she began experiencing a slowly progressive decline in cognitive function, with impairments in memory and learning, and executive domains. Extensive investigation did not reveal a reversible cause for the cognitive decline. At the age of 55, she had an episode of stroke, resulting in left hemiparesis. After that event, we diagnosed SS, leading to the initiation of warfarin, after which she experienced no recurrence of cerebrovascular events in the follow-up of nine years.

In addition, her brain MRI revealed significant cerebral atrophy with disproportionate ventricular dilation. Despite this finding, she did not have intracranial hypertension, and the tap test did not lead to an improvement in cognitive symptoms. She tested positive for ACL and LA.

## Case 2

This woman experienced three episodes of cerebrovascular events when she was 19 and 20 years old. In the first episode, she presented with monoparesis of the right upper limb. The second episode was characterized by paresis of the lower segment of the right face. Her last episode involved motor deficit in the left upper limb. MRI performed 14 years after the last event revealed areas of encephalomalacia in the right perirolandic region, in the left middle frontal gyrus, and in the middle thirds of the cerebellar hemispheres. Her dermatological evaluation revealed, in addition to LR, the presence of Raynaud’s phenomenon. She also had episodic tension-type headaches. She had one pregnancy, without complications. Further investigation for thrombophilias and autoimmune diseases was negative. Until the third event, she had not initiated any form of antithrombotic therapy, as she was initially diagnosed with multiple sclerosis, due to suggestive MRI findings and the absence of cardiovascular risk factors, rather than ischemic stroke. After being diagnosed with SS, anticoagulant therapy with warfarin was started, and the patient experienced no further cerebrovascular events in the follow-up of eight years.

## Case 3

A 39-year-old woman presented with slurred speech and right-sided hemiparesis. Magnetic resonance imaging (MRI) revealed ischemia in the left internal capsule territory. Dermatological examination showed the presence of LR. This woman had a prior diagnosis of SLE, with cutaneous and articular symptoms, treated with hydroxychloroquine and azathioprine. Additionally, she had a history of episodic migraine with aura since her adolescence, as well as systemic arterial hypertension. In her obstetric history, she had a record of two miscarriages. APS antibodies were all negative. She started on warfarin and did not experience subsequent cerebrovascular events in seven years of follow-up.

## Case 4

A 31-year-old woman presented with a sudden onset of global cerebellar ataxia. MRI revealed ischemia in the right cerebellar hemisphere, in the territory of the anterior inferior cerebellar artery. This woman also had a history of gravidic chorea. She had a history of migraines with visual aura. Besides the LR, she had tested positive for antinuclear antibody (ANA) with a coarse speckled pattern (titer > 1:640) and positive Aβ2GP1. Despite the presence of high-titer ANA, further investigation did not lead to the diagnosis of another autoimmune pathology. She had not had miscarriages or other relevant medical history. Anticoagulant therapy with warfarin was initiated, and in the follow-up of three years she has not had any new cerebrovascular events.

## Case 5

A 26-year-old woman presented with a sudden onset of left-sided hypoesthesia. MRI showed an acute ischemia in the right postcentral gyrus. Her dermatological examination revealed, in addition to LR, the presence of Raynaud’s phenomenon. Anticardiolipin (ACL), lupus anticoagulant (LA), and Aβ2GP were positive. She also had a history of migraines with aura. Due to the diagnosis of SS and APS, with positivity for all three autoantibodies, a dual therapy with warfarin and acetylsalicylic acid was chosen. The patient did not experience new thrombotic events in the follow-up of four years.

## Case 6

**Fig. 2:**
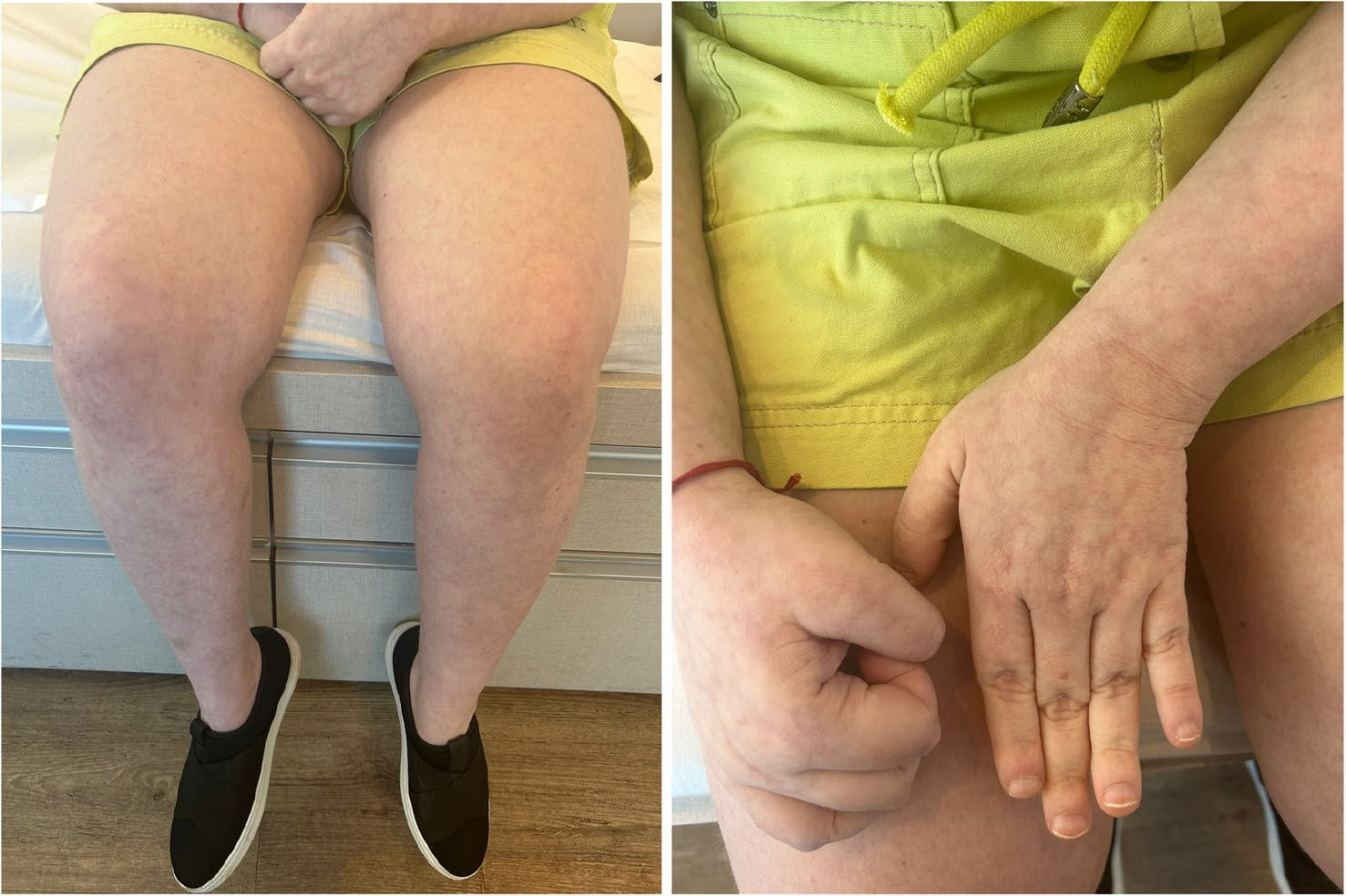
From Patient #6. Right: Erythematous lesions of varying shapes and sizes, with a diffuse and relatively uniform distribution consistent with LR, are observed on the patient’s thighs and knees. Left: Lesions with the same pattern are seen on the patient’s hands and forearms

A 22-year-old woman presented with a sudden onset of aphasia and right-sided hemiparesis. MRI showed high signal in diffusion-weighted imaging in the territory of the left middle cerebral artery. Afterwards, she developed focal epileptic seizures, including an episode of status epilepticus. Her epilepsy was controlled with an association of lacosamide, topiramate and clobazam. She had a pre-existing history of SLE, treated with prednisone and azathioprine. Additionally, she had antithrombin III deficiency. She was nulliparous, and all serologies for APS were negative. Her dermatologic examination revealed LR (Fig. 2). Upon diagnosis of SS, anticoagulant therapy with warfarin was initiated, and the patient experienced no new cerebrovascular events in the follow-up of two years.

## Discussion

SS is a rare disorder that primarily affects women, with the diagnosis typically confirmed between the third and fifth decades of life, often following a cerebrovascular event [3], in line with our cases. Cutaneous manifestations may precede neurological symptoms by more than 10 years, often going unnoticed by patients, thereby indicating the onset of the disease long before a diagnosis is established [4]. While typically sporadic, familial cases with an autosomal dominant genetic inheritance pattern have been reported [5].

SS may be associated with APS antibodies (lupus anticoagulant, anti-beta 2 glycoprotein 1, and anticardiolipin) is frequently reported. In a comparative study of 46 patients with SS, 19 (41%) tested positive for one of these antibodies [6]. This association has led authors to classify SS as either primary or secondary. Secondary SS occurs in the presence of a defined thrombophilia, such as APS [7].

In Table 2 we present demographic characteristics, neurological manifestations, and major comorbidities reported in case series.

**Table 2:** Clinical characteristics of SS patients in published reports

Author	*n*	Female	Neurological features			Comorbidities				
			Headache, type	Epilepsy	Cognitive dysfunction	Positive APSa	Other AI disease	Thrombophilia	SAH	Heart valve involvement
Zelger, 1993	21	86	MA (35), other (53)	29	MCI (48), dementia (10)	0	NR	NR	62	Aortic valve insufficiency (10)
Boesch, 2003	13	92	Migraine (15), other (69)	NR	Subjective cognitive decline (77)	0	NR	Protein S deficiency (23)	15	NR
Bolayir, 2004	10	100	MA (30)	20	NR	60	Myasthenia gravis (10)	AT III deficiency (10)	40	MV stenosis (20)
Bottin, 2015	53	83	Migraine (25), other (25)	16	MCI (60), dementia (3)	0*	NR	NR	55	LSV (19), leaflet thickening (26)
Yao, 2020	7	43	NR	29	Unspecified (71)	71	NR	NR	100	Unspecified (43)
Yilmaz, 2021	12	92	NR	33	Dementia (17)	0	NR	MTHFR (50)	42	MV insufficiency (50), MV thickening (33)
Current report	6	100	MA (50), tension type (17)	33	Dementia (17)	50	SLE (33), Raynaud’s (33)	AT III deficiency (17)	33	MV thickening (17), MV insufficiency (17)

Numeric data means %, except for n column, which means absolute numbers

*ACL* Anticardiolipin antibodies, *AI* Autoimmune, *APSa* Antiphospholipid syndrome antibodies, *AT III* Antithrombin III, *anti Aβ2GP1* Anti-beta2-glycoprotein 1 antibodies, *LA* Lupus anticoagulant, *LSV* Libman-Sacks vegetations, *MA* Migraine with aura, *MCI* Mild cognitive impairment, *MTHFR* Methylene-tetra-hydrofolate-reductase, *MV* Mitral valve, *SAH* Systemic arterial hypertension, *SLE* Systemic lupus erythematosus

*This study was specifically designed to report SS cases with no APS antibodies.

### Dermatological manifestations

LR consists of purplish, irregular, semicircular streaks that initially appear in the lower lumbar region and buttocks, later involving the extensor surface of the arms and thighs. The upper lumbar region and extremities are affected only in more extensive cases. Although LR is characteristically a persistent skin alteration, its extent or intensity may increase when the skin is exposed to colder temperatures [4]. Thromboses in dermal arterioles, supplied by circular segments of the skin, trigger venular dilatation in the same territory. This generates the violaceous coloration of LR [8].

Skin biopsies, which are not mandatory for diagnosis, reveal involvement of small and medium-sized arteries, which begins as T-cell-mediated endothelial inflammation, followed by subendothelial proliferation of smooth muscle cells and extracellular matrix [9, 10].

### Neurological manifestations

Cerebrovascular events typically occur around the fourth decade and are most often ischemic stroke, caused by atherothrombosis. The most frequent neurological symptoms are sudden motor deficits, followed by changes in speech or language (dysarthria and aphasia) and visual defects. Eye movement abnormalities and sensory, cerebellar and vestibular symptoms are observed less frequently. Epileptic seizures have also been reported, with patients with APS antibodies appearing to be more susceptible to this manifestation [6], although it was not what we found.

Hemorrhagic strokes in patients with SS have been reported, not exclusively in patients who were undergoing antiplatelet or anticoagulant therapy following previous ischemic strokes. Patients with intraparenchymal hemorrhage were described, probably related to vascular abnormalities, similar to what is observed in Moyamoya disease [11, 12].

Headache can be the initial neurological manifestation in patients with SS, and the predominant phenotype is migraine [4], in accordance with our cases. Accordingly, the presence of LR is more common in patients with migraines, and in this group, it constitutes an independent risk factor for the occurrence of stroke [13, 14]. Although migraine is also a common manifestation of APS, the presence of migraines occurs independently of positivity for APS antibodies in patients with SS [15].

Cognitive decline typically arises in advanced stages, resulting from multiple cerebral small vessel ischemia and subsequent cerebral atrophy. Such impairment may manifest as language deficits, visuospatial disorientation, and memory loss [16]. The relatively young age and the short follow-up period since the stroke may explain the absence of cognitive symptoms in most patients in our case series. In certain cases, as in case #1, cognitive symptoms can be the initial manifestation of the disease, in an age group not typical for degenerative causes of dementia. In this context, SS becomes a differential diagnosis in cases of early-onset dementia [17, 18].

In terms of neuroimaging findings, the most frequently observed results on MRI include cortical and subcortical ischemias of medium or small size, cerebellar ischemias, and diffuse cerebral atrophy, with a potentially higher degree of atrophy in the areas affected by ischemia. Ischemias involving large arterial territories are not expected. Deep white matter lesions suggestive of leukoaraiosis may also be found, especially in hypertensive patients [19, 20].

More recently, cerebral microbleeds and chronic superficial siderosis have also been described, and due to their location coinciding with areas of greater cortical atrophy, they have been suggested as an additional mechanism for the cognitive decline in these patients [21].

Angiography is not necessary to establish the diagnosis, but it usually reveals stenoses and occlusions of medium-sized intracranial arteries, and in some cases, a pattern of pseudoangiomatosis [19, 20].

Psychiatric symptoms, absent in our cases, have rarely been reported as the main clinical presentation, with reported cases including severe depression and hypomanic episodes leading to the diagnosis of bipolar II disorder; psychosis with auditory hallucinations; and persecutory delusions associated with visual, auditory hallucinations, and suicide attempts [22, 23].

### Treatment

The treatment aims to reduce the risk of recurrent ischemic cerebrovascular events. It can be carried out with antiplatelet or anticoagulant therapy, with currently no evidence supporting a preference for one therapy over the other. The presence of a comorbidity that indicates a specific treatment, such as anticoagulation in patients with atrial fibrillation, may guide the decision. In a retrospective study, patients with APS have been reported to be at a higher risk of stroke recurrence under antiplatelet therapy compared to anticoagulant therapy (0.5 versus 0.06 recurrent cerebral events per year) [6]. Another study found a similar annual recurrence rate of cerebrovascular events in both antiplatelet (3.0%) and anticoagulant (2.7%) therapy groups in patients with no APL antibodies [24].

We chose to treat all our patients with anticoagulation using warfarin, targeting an INR of 2 to 3, with no episodes of stroke recurrence to date. In case #5, the patient, who tested positive for all APS antibodies, was treated with both anticoagulation and antiplatelet therapy.

## Conclusion

It is important for clinicians to recognize SS as a possible etiology of stroke, particularly in young women. A thorough dermatological examination can aid in early detection and may change the course of treatment, especially when other underlying conditions such as APS or SLE are present.

## Data Availability

This study is a case series and does not include datasets. Additional clinical information related to the cases presented is available from the corresponding author upon reasonable request and subject to appropriate ethical considerations.

## References

[1] Wu S, Xu Z, Liang H. Sneddon’s syndrome: a comprehensive review of the literature. Orphanet J Rare Dis. 2014;9:215. 25551694 10.1186/s13023-014-0215-4 PMC4302600

[2] Sneddon IB. Cerebro-vascular lesions and Livedo reticularis. Br J Dermatol. 1965;77(4):180–5. 14278790 10.1111/j.1365-2133.1965.tb14628.x

[3] Ertugrul B, et al. Sneddon’s syndrome: clinical and laboratory analysis of 10 cases. Acta Med Okayama. 2004;58(2):59–65. 15255506 10.18926/AMO/32100

[4] Zelger B, Sepp N, Stockhammer G, et al. Sneddon’s syndrome: A Long-term Follow-up of 21 patients. Arch Dermatol. 1993;129(4):437–47. 8466214 10.1001/archderm.129.4.437

[5] Mascarenhas R, et al. Familial sneddon’s syndrome. Eur J Dermatology. 2003;13(3):283–7. 12804991

[6] Francès C, et al. Sneddon syndrome with or without antiphospholipid antibodies. A comparative study in 46 patients. Medicine (Baltimore). 1999;78(4):209–19. 10424203 10.1097/00005792-199907000-00001

[7] Schellong SM. Classification of sneddon’s syndrome. Vasa - J Vascular Dis. 1997;26(3):215–21. 9286155

[8] Samanta D, Cobb S, Arya K. Sneddon syndrome: A comprehensive overview. J Stroke Cerebrovasc Dis. 2019;28(8):2098–108. 31160219 10.1016/j.jstrokecerebrovasdis.2019.05.013

[9] Sepp N, Zelger B, Schuler G, Romani N, Fritsch P. Sneddon’s syndrome–an inflammatory disorder of small arteries followed by smooth muscle proliferation. Immunohistochemical and ultrastructural evidence. Am J Surg Pathol. 1995;19(4):448–53. 7694946 10.1097/00000478-199504000-00006

[10] Lewandowska E, et al. Sneddon’s syndrome as a disorder of small arteries with endothelial cells proliferation: ultrastructural and neuroimaging study. Folia Neuropathol. 2005;43(4):345–54. 16416398

[11] de Aquino Gondim FA, Leacock RO, Subrammanian TA, Cruz-Flores S. Intracerebral hemorrhage associated with Sneddon’s syndrome: is ischemia-related angiogenesis the cause? Case report and review of the literature. Neuroradiology. 2003June;45(6):368–72. 12750866 10.1007/s00234-003-0990-4

[12] Killeen T, Wanke I, Mangiardi J, Cesnulis E. Ruptured, fusiform, distal lenticulostriate aneurysm causing intraventricular haemorrhage in a patient with antiphospholipid-negative Sneddon’s syndrome. Clin Neurol Neurosurg. 2014;116:80–2. 24300743 10.1016/j.clineuro.2013.11.009

[13] Tietjen GE, Al-Qasmi MM, Shukairy MS. Livedo reticularis and migraine: a marker for stroke risk? Headache. 2002;42(5):352–5. 12047335 10.1046/j.1526-4610.2002.02106.x

[14] Tietjen GE, Gottwald L, Al-Qasmi MM, Gunda P, Khuder SA. Migraine is associated with Livedo reticularis: a prospective study. Headache. 2002;42(4):263–7. 12010382 10.1046/j.1526-4610.2002.02078.x

[15] Tietjen GE, Al-Qasmi MM, Gunda P, Herial NA. Sneddon’s syndrome: another migraine-stroke association? Cephalalgia. 2006;26(3):225–32. 16472327 10.1111/j.1468-2982.2005.01032.x

[16] Weissenborn K, et al. Neuropsychological deficits in patients with Sneddon’s syndrome. J Neurol. 1996;243(4):357–63. 8965111 10.1007/BF00868412

[17] Fabiani G, et al. Cognitive and psychiatric changes as first clinical presentation in Sneddon syndrome. Dement Neuropsychol. 2018;12(2):216–9. 29988314 10.1590/1980-57642018dn12-020016 PMC6022978

[18] Wright RA, Kokmen E. Gradually progressive dementia without discrete cerebrovascular events in a patient with Sneddon’s syndrome. Mayo Clin Proc. 1999;74(1):57–61. 9987534 10.4065/74.1.57

[19] Yilmaz E, Arsava EM, Gocmen R, Oguz KK, Arat A, Topcuoglu MA. Characteristic imaging features of neurovascular involvement in primary Sneddon’s syndrome: an analysis of 12 cases. Neurol Sci. 2021;42(6):2363–9. 33047201 10.1007/s10072-020-04621-0

[20] Boesch SM, Plörer AL, Auer AJ, Poewe W, Aichner FT, Felber SR, Sepp NT. The natural course of Sneddon syndrome: clinical and magnetic resonance imaging findings in a prospective six year observation study. J Neurol Neurosurg Psychiatry. 2003;74(4):542–4. 12640088 10.1136/jnnp.74.4.542 PMC1738396

[21] Yao M, Zhao J, Jiang N, Li L, Ni J. Superficial siderosis and microbleed restricted in cortex might be correlated to atrophy and cognitive decline in sneddon’s syndrome. Front Neurol. 2020;11:1035. 33041979 10.3389/fneur.2020.01035 PMC7525095

[22] Lalanne L, et al. A suicide attempt in a context of bipolar symptoms leading to a diagnosis of Sneddon syndrome. J Neuropsychiatry Clin Neurosci. 2013;25(3):E11–2. 24026725 10.1176/appi.neuropsych.11120360

[23] Hsu FF, Chung KH. Psychosis with suicide attempt in Sneddon syndrome. Psychiatry Clin Neurosci. 2017;71(2):147–8. 27891710 10.1111/pcn.12489

[24] Bottin L, et al. Strokes in Sneddon syndrome without antiphospholipid antibodies. Ann Neurol. 2015;77(5):817–29. 25628239 10.1002/ana.24382

